# Quantitative analysis of *SOD2*, *ALDH1A1* and *MGST1* messenger ribonucleic acid in anterior lens epithelium of patients with pseudoexfoliation syndrome

**Published:** 2013-06-12

**Authors:** Barbara Strzalka-Mrozik, Lilianna Prudlo, Malgorzata W. Kimsa, Magdalena C. Kimsa, Malgorzata Kapral, Malgorzata Nita, Urszula Mazurek

**Affiliations:** 1 Department of Molecular Biology, Medical University of Silesia, Sosnowiec, Poland; 2 Department of Ophthalmology, Medical University of Silesia, Katowice, Poland; 3 Department of Biochemistry, Medical University of Silesia, Sosnowiec, Poland

## Abstract

**Purpose:**

The aim of the study was to investigate the expression of selected genes encoding enzymes involved in the antioxidant defense system (superoxide dismutase 2, *SOD2*; aldehyde dehydrogenase 1, *ALDH1A1*; microsomal glutathione S-transferase 1, *MGST1*) in fragments of anterior lens capsules of patients with pseudoexfoliation syndrome (PEX). The specificity and sensitivity of these molecular markers for PEX development were also assessed.

**Methods:**

The study group consisted of 20 patients (9 women and 11 men) with diagnosed PEX and cataract. The control group included 23 patients (8 women and 15 men) who needed cataract surgery but did not have PEX. Quantification of *SOD2*, *ALDH1A1*, and *MGST1* messenger ribonucleic acid (mRNA) was performed with quantitative real-time PCR.

**Results:**

*SOD2*, *ALDH1A1*, and *MGST1* mRNAs were detected in all studied samples. The examined genes had statistically significant higher expression in the group of patients with PEX than in the control group (*SOD2*, p=0.0015; *ALDH1A1*, p=0.0001; *MGST1*, p=0.0001, Mann–Whitney U test). The areas under the curve (AUC) of *SOD2*, *MGST1*, and *ALDH1A1* were 0.766, 0.818, and 0.957, respectively.

**Conclusions:**

Differential expression of *SOD2*, *ALDH1A1*, and *MGST1* genes in the anterior lens capsules of patients with PEX suggest that diseased tissue appears to respond to the previously reported oxidative stress. A possible role of *ALDH1A1* mRNA level as a risk factor or marker for PEX needs further confirmation.

## Introduction

Pseudoexfoliation syndrome (PEX) is a serious ocular manifestation of a common age-related systemic disorder, and is frequently associated with severe chronic secondary open-angle glaucoma and cataract [1]. PEX involves pathologic excessive production, abnormal crosslinking [2,3], and progressive accumulation of elastic microfibrils in many organs [4,5].

Several mechanisms of the pseudoexfoliation material formation are possible, but a molecular background is more frequently suggested [6]. The imbalance between the antioxidant defense mechanism and reactive oxygen species (ROS) production results in oxidative stress leading to cellular damage. In addition, oxidative stress mechanisms (e.g., exposure to environmental chemicals, radiation, and atmospheric oxygen) in ocular tissues promotes various pathological conditions, including eye diseases such as cataract, glaucoma, uveitis, and age-related macular degeneration [7-9]. The cellular antioxidant defense system comprises enzymatic (i.e., superoxide dismutases [SODs], catalase, glutathione peroxidase) and non-enzymatic antioxidants (i.e., vitamins A, C, and E, selenium, zinc, carotenoids, and various metabolites) [10-13]. Moreover, Lobo et al. [14] and Sies [15] distinguished three levels of the antioxidant defense system. The first line contributes to preventing formation of free radicals and comprises enzymes such as superoxide dismutase, catalase, and glutathione peroxidase. The second line of defense contributes to suppressing chain initiation and/or breaking the chain propagation reactions and involves the low-molecular-weight antioxidants, i.e., vitamins and enzymes involved in its pathway such as aldehyde dehydrogenase 1 (*ALDH1A1*). The third line of defense is composed of a repair enzyme such as glutathione peroxidase and microsomal glutathione S-transferase 1 (*MGST1)* [14,15].

ROS are formed as normal metabolic products and are important in normal cellular functioning, but their production can be increased under pathological conditions and cause damage [4,5]. The generation of ROS can cause lipid peroxidation, protein modification and denaturation, and DNA damage [16]. Therefore, numerous antioxidant systems act as protective mechanisms. The type of antioxidant used in the free radical reaction depends on the site of free radical generation and its properties. The main ROS molecules that generate in the lens include superoxide anion, hydroxyl radical, and H_2_O_2_ [17]. Growing evidence supports the importance of oxidative stress in PEX [3-5,18]. Understanding the role of oxidative stress in the development of PEX may provide novel opportunities for improved therapeutic intervention. However, the exact pathogenesis of this disorder has not been well understood yet. Oxidative stress may be determined through markers of lipid peroxidation, the activity of antioxidant enzymes, and the levels of low-molecular-weight antioxidants [9]. We proposed single genes as components of three levels of the antioxidant defense system for the activity analysis of those lines. Therefore, the present study focused on quantitative relationships of *SOD2*, *ALDH1A1*, and *MGST1* messenger ribonucleic acid (mRNA) levels between patients presenting with cataracts with and without PEX. Additionally, the specificity and sensitivity of these molecular markers for PEX development were assessed.

## Methods

### Subjects

The forty three patients with PEX and cataract or with cataract alone participated in this study (17 women and 26 men, mean age 73 years; range 63-80 years). The examined group comprised 20 patients (9 women and 11 men, mean age 74.5 years; range 63–80 years) with clinically diagnosed PEX and cataracts. The control group consisted of 23 cataract patients (8 women and 15 men, mean age 71 years; range 66–80 years) without PEX (Table 1). All the patients were treated at the Department of Ophthalmology, University Hospital No. 5, Medical University of Silesia, Katowice, Poland.

**Table 1:** Selected clinical features of patients with clinically diagnosed PEX syndrome and the control group.

Characteristic	PEX syndrome group (n=20)	Control group (n=23)
Gender		
Female	9	8
Male	11	15
Age (years)	74.5 (63 - 80) *	71 (66 - 80)
Eye		
Right	9	15
Left	11	8
Visual acuity ^†^	0.1 (0.01 – 0.5)	0.2 (0.01–0.7)
Axial length (mm)	23.11 (22.34 – 24.98)	23.52 (22 – 27)
IOP (mmHg) ^‡^	17 (16 – 20)	17 (16 - 21)

* Values of clinical parameters are expressed as means (minimum-maximum) ^†^ Snellen chart ^‡^ IOP - intraocular pressure

All subjects underwent a complete ophthalmic examination: best-corrected visual acuity (BCVA) using a 6-m Snellen chart, Goldmann applanation tonometry, gonioscopy with the use of three mirror Goldmann goniolens in the primary position of gaze, and direct and indirect slit-lamp biomicroscopy after pupil dilation with 1% Tropicamide (Haag-Streit, Köniz, Switzerland, +78D Oculus). The presence of PEX was confirmed clinically by PEX material deposits on anterior segment structures on the pupillary margin, anterior lens capsule (ALC), corneal endothelium, and various pigment-related signs—loss of pigment at the pupillary ruff, irregular pigmentation of trabecular meshwork, and the accumulation of pigment along Schwalbe’s line (Sampaolesi’s line).

The criteria for inclusion in the molecular analysis were as follows: aged ≥60 years, PEX exfoliation material on anterior segment structures, and scheduled for routine phacoemulsification cataract surgery. Patients with a history of previous intraocular surgery, glaucoma, or any other systemic or ocular conditions were excluded from the study. In addition, we excluded patients with no known history or symptoms of diabetes or other chronic disease, which could affect the measurements.

The study was approved by the Bioethics Committee of the Medical University in Katowice (KNW-6501–12/I/08) in accordance with the Declaration of Helsinki regarding medical research involving human subjects. All patients were informed about the research and signed an informed consent form.

### Ribonucleic acid extraction from tissue specimens

Circular sections of ALCs from 20 eyes with PEX and cataract and 23 eyes with cataract alone were collected during phacoemulsification cataract surgery. The sections were stored for 48 h at −70 °C until RNA extraction. Total RNA was extracted from ALCs using a TRIzol reagent (Invitrogen, Carlsbad, CA). RNA extracts were treated with DNase I (MBI Fermentas, Vilnius, Lithuania) according to the manufacturer’s instructions. The quality of the extracts was checked electrophoretically using 0.8% agarose gel stained with ethidium bromide. The results were analyzed and recorded using the 1D Bas-Sys gel documentation system (Biotech-Fisher, Perth, Australia). Total RNA concentration was determined with spectrophotometric measurement in 5 μl capillary tubes using the Gene Quant II RNA/DNA Calculator (Pharmacia Biotech, Cambridge, UK).

### Quantitative real-time polymerase chain reaction assay

Detection of the expression of *SOD2*, *ALDH1A1*, *MGST1*, and glyceraldehyde-3-phosphate (*GAPDH*) mRNAs was performed using a quantitative real-time (RT) PCR with SYBR Green chemistry (SYBR Green Quantitect RT–PCR Kit, Qiagen, Valencia, CA) according to the manufacturer’s instructions, and an Opticon DNA Engine Continuous Fluorescence detector (MJ Research, Waltham, MA) as described previously [19]. All samples were tested in triplicate. GAPDH for each sample was measured to exclude possible RT-PCR inhibitors. Oligonucleotide primers, specific for *SOD2* [20], *ALDH1A1* [21], *MGST1* [18], and *GAPDH* [19], were chosen based on the published data (Table 2). The thermal profile for one-step RT-PCR was as follows: reverse transcription at 50 °C for 30 min, denaturation at 95 °C for 15 min, and 40 cycles consisting of the following temperatures and time intervals, 94 °C for 15 s, 60 °C for 30 s, and 72 °C for 30 s. A cycle threshold (Ct), which is the point at which a PCR product is for the first time detected above a fixed threshold, was determined for each sample.

**Table 2:** Characteristics of primers used for real-time QRT-PCR

Gene	Sequence of primers	Length of amplicon (bp) *	Tm (°C) ^†^
SOD2	Forward: 5′-CTGATTTGGACAAGCAGCAA-3′	199	81.6
	Reverse: 5′-CTGGACAAACCTCAGCCCTA-3′		
ALDH1A1	Forward: 5′-TACTCACCGATTTGAAGATT-3′	151	77.2
	Reverse: 5′-TTGTCAACATCCTCCTTATC-3′		
MGST1	Forward: 5′- ATTGGCCTCCTGTATTCCTTG-3′	311	80.2
	Reverse: 5′-TAATCCCTCTGCTCCCCTCC-3′		
GAPDH	Forward: 5′-GAAGGTGAAGGTCGGAGTC-3′	226	80.0
	Reverse: 5′-GAAGATGGTGATGGGATTC-3′		

* bp – base pairs ^†^ Tm – melting temperature

To quantify the results obtained with RT-PCR for *SOD2*, *ALDH1A1*, *MGST1*, and *GAPDH*, a standard curve method was used [22,23]. Commercially available standards of β-actin cDNA (TaqMan DNA Template Reagent Kit, PE Applied Biosystems, Foster, CA) were used at five different concentrations (0.6, 1.2, 3.0, 6.0, and 12.0 ng/µl), to simultaneously detect the expression profile of each investigated gene. For standards, the calculation of copy number values was based on the following relationship: 1 ng of DNA=333 genome equivalents (PE Applied Biosystems). Amplification plots for each dilution of a commercially available standard template were used to determine the Ct values. A standard curve was generated by plotting the Ct values against the log of the known amount of β-actin cDNA copy numbers. Correlation coefficients for standard curves ranged from 0.988 to 0.995 indicating a high degree of confidence for measurement of the copy number of molecules in each sample.

Each run was completed using melting curve analysis to confirm the specificity of amplification and the absence of primer dimers. RT-PCR products were separated on 6% polyacrylamide gels and visualized with silver salts.

### Statistical analyses

Statistical analyses were performed using Statistica 8.0 software (StatSoft, Tulsa, OK), and the level of significance was set at p<0.05. Values were expressed as median with the 25th and 75th quartiles, and minimum and maximum. The Mann–Whitney U test was applied to assess differences in the expression of *SOD2*, *ALDH1A1*, and *MGST1*.

Respective cutoff values for gene expression were assessed with the receiver operating characteristic (ROC) curve analysis. With the use of ROC analysis, sensitivities and specificities were calculated by varying the criterion of positivity from the least (cut at probability of 0) to the most stringent (cut at probability of 1). Optimal sensitivity and specificity were determined for the *SOD2*, *MGST1*, and *ALDH1A1* genes, and a corresponding cutoff value of each gene was identified. The area under the curve (AUC) of each gene and  a relative risk of PEX development were also calculated. The AUC has a value from 0.5 to 1.0, where 1.0 represents perfect ability to discriminate, and 0.5 represents the discrimination resulting from pure chance An AUC greater than 0.9 is considered excellent, greater than 0.8 to 0.9 very good, 0.7 to 0.8 good, 0.6 to 0.7 average, and <0.6 poor [24,25].

## Results

### Specificity of the real-time polymerase chain reaction assay

RT-PCR specificity for the target genes was confirmed experimentally based on amplimers melting temperatures. For each RT-PCR product, a single peak at the expected temperatures was observed: *SOD2*, 81.6 °C; *ALDH1A1*, 77.2 °C; *MGST1*, 80.2 °C; and *GAPDH*, 80.0 °C (data not shown). Gel electrophoresis also revealed the presence of a single product of predicted length (data not shown).

### The messenger ribonucleic acid level of superoxide dismutase 2, aldehyde dehydrogenase 1, and microsomal glutathione S-transferase 1 in anterior lens capsules of patients with pseudoexfoliation syndrome

**Figure 1:**
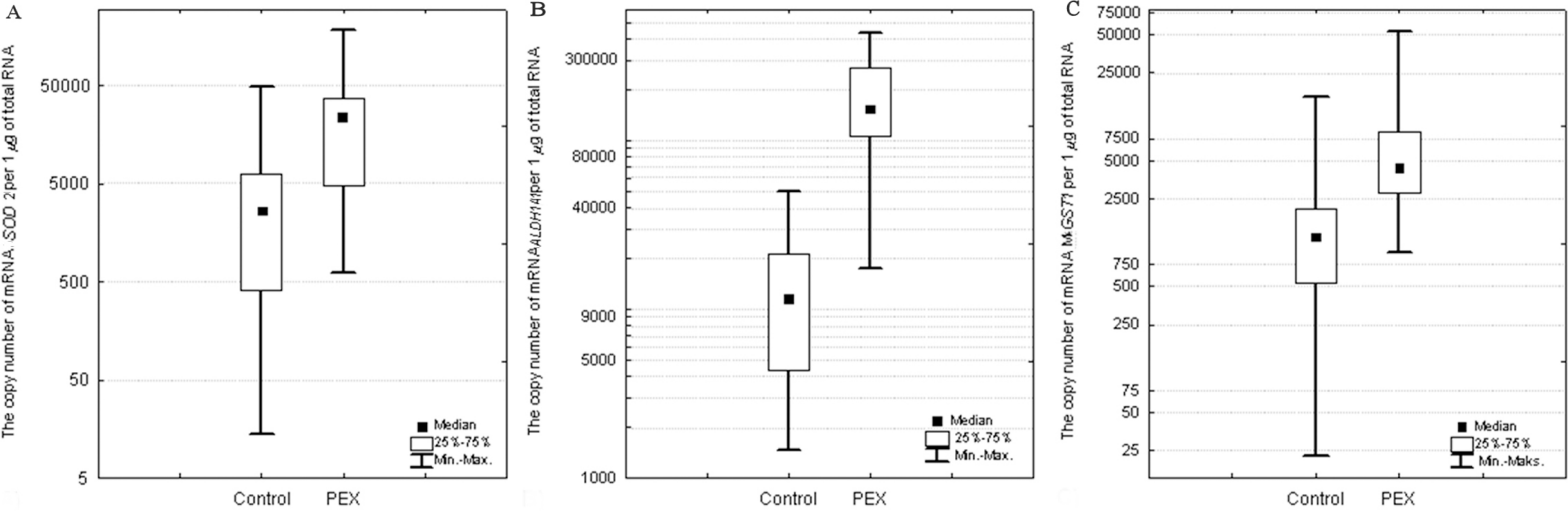
The messenger ribonucleic acid (mRNA) level of superoxide dismutase 2 (*SOD2*; **A**), aldehyde dehydrogenase 1 (*ALDH1A1*; **B**), and microsomal glutathione S-transferase 1 (*MGST1*; **C**) in the ALCs of patients with clinically diagnosed pseudoexfoliation syndrome (PEX) and in the control group. (Box and whisker plots present medians±quartiles and extreme values of copy numbers per 1 μg of total RNA; p<0.05, Mann–Whitney U test.)

*SOD2*, *ALDH1A1*, and *MGST1* mRNAs were detected in all tested samples obtained from the control and study groups. In the ALCs, the *SOD2* mRNA copies/µg of total RNA was about ninefold higher in the patients with PEX and cataract (median=23,288 mRNA copies/µg of total RNA) compared to the control cataract group (median=2,600 mRNA copies/µg of total RNA), and statistical significance was found (p=0.0015, Mann–Whitney U test; Figure 1A). In the case of *ALDH1A1* mRNA, there was a statistically significant 13-fold increase in the study group (median=153,001 mRNA copies/µg of total RNA) compared to the control group (median=11,598 mRNA copies/µg of total RNA; p=0.0001, Mann–Whitney U test; Figure 1B). The mRNA level of *MGST1* was about threefold higher in the PEX and cataract samples (median=4,439 mRNA copies/µg of total RNA) than in the control cataract group samples (median=1,243 mRNA copies/µg of total RNA). A comparative analysis of *MGST1* expression also revealed statistically significant differences between the patients with PEX and cataract and the control cataract groups (p=0.0001, Mann–Whitney U test; Figure 1C).

### Assessment of superoxide dismutase 2, aldehyde dehydrogenase 1, and microsomal glutathione S-transferase 1 messenger ribonucleic acid level as molecular markers of pseudoexfoliation syndrome

**Figure 2:**
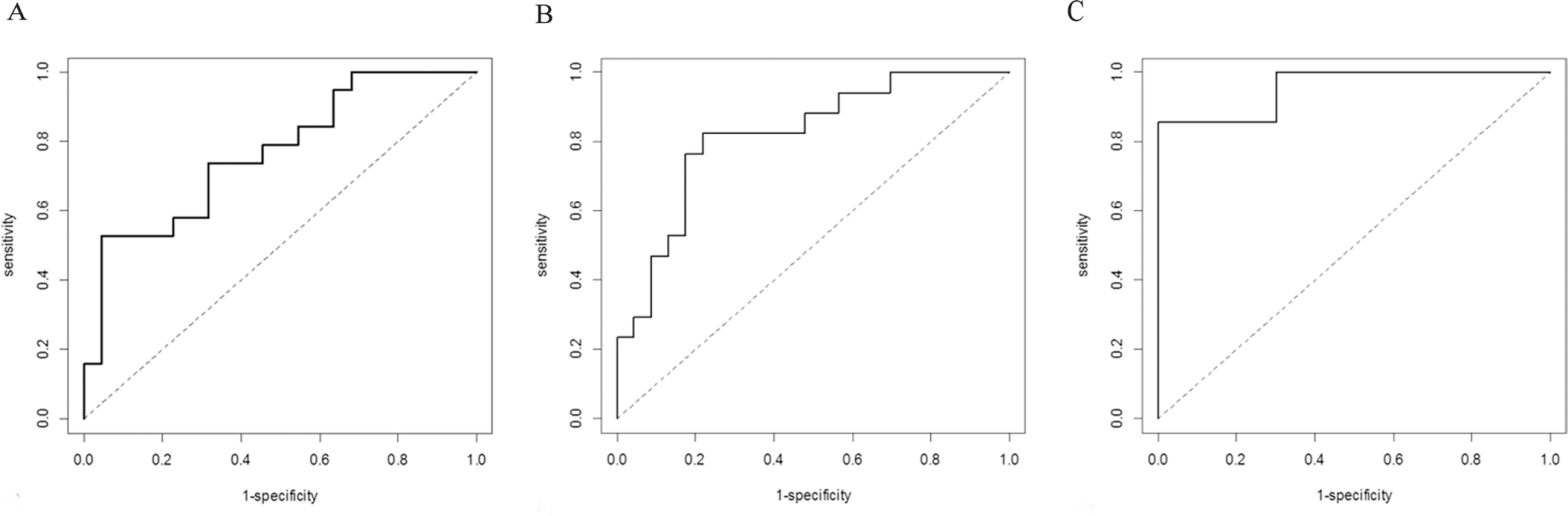
The receiver operating characteristic (ROC) curves for superoxide dismutase 2 (*SOD2*; **A**), aldehyde dehydrogenase 1 (*ALDH1A1*; **B**), and microsomal glutathione S-transferase 1 (*MGST1*; **C**) mRNA levels display molecular markers of pseudoexfoliation syndrome (PEX).

To evaluate the predictive power of *SOD2*, *MGST1*, and *ALDH1A1* mRNA levels as potential molecular markers of PEX development, the AUC, sensitivity, specificity, and cutoff value were shown with use of the ROC curve analysis (Table 3). The *SOD2* mRNA level above 23,288 copies/µg of total RNA and the *MGST1* mRNA level above 2,155 copies/µg of total RNA were associated with a 1.7-fold and 1.8-fold increase in the relative risk of PEX development, respectively. The cutoff value for the *ALDH1A1* gene (mRNA level above 67,000 copies/µg RNA) corresponded to a 2.4-fold increase in a relative risk of PEX development. The AUCs of *SOD2*, *MGST1*, and *ALDH1A1* were 0.766, 0.818, and 0.957, respectively, and all were higher than 0.7 (p<0.001; Figure 2A–C).

**Table 3:** The ROC analysis and the diagnostic values of the *SOD2*, *MGST1* and *ALDH1A1* mRNA levels.

Variable	AUC *	cutoff value	specificity [%]	sensitivity [%]
		(**mRNA copies/µg RNA)**		
SOD2	0.766	23,288	95.5%	52.6%
MGST1	0.818	2155	78.3%	82.4%
ALDH1A1	0.957	67,000	100.0%	85.7%

* AUC - area under the curve

## Discussion

The pathogenesis of PEX seems to be multifactorial but remains unexplained [26]. PEX can be associated with a disturbed extracellular matrix synthesis, uncontrolled basement membrane metabolism, and the action of free radicals [27]. Moreover, the oxidant-antioxidant balance may be altered in various ocular pathologies such as cataract, glaucoma, uveitis, and age-related macular degeneration [12,28-31]. Oxidative stress leads to damage to lipids and DNA and the inhibition and deactivation of proteins such as collagen and elastin resulting in disruption of overall biologic function [27,32,33]. At the cell membrane, lipid hydroperoxides induce changes in permeability and cause an uncoupling of the membrane-bound enzyme sodium, potassium-adenosine triphosphatase (Na,K-ATPase) and oxidative inhibition of calcium-adenosine triphosphatase (Ca^2+^-ATPase) in several tissues, including the lens. The decreased activity of these enzymes is thought to be connected to oxidative damage to the sulfhydryl groups of the molecules. In turn, within the cell, lipid peroxides can damage DNA [34]. Gartaganis et al. [27] confirmed an increase in lipid peroxidation in PEX lens epithelial cells.

The antioxidants in the defense systems act at different levels such as preventive, radical scavenging, and repair. In the present study, qRT-PCR was applied to investigate the *SOD2*, *ALDH1A1*, and *MGST1* mRNA levels encoding selected enzymes, which are antioxidants acting in the defense systems at all three levels.

In previous research, antioxidant activity changed due to eye diseases in various ocular tissues such as the aqueous humor, the conjunctiva, the cornea, and the lens [27,35-39]. Many attempts have also been made to identify a total antioxidant status and concentrations of individual antioxidant enzymes in serum, which can be easily assessed [28,29,40-42]. However, the systemic alterations in antioxidants might not exactly reflect the situation in the structures of eyes [28]. In addition, the change in the protein level is preceded by alteration of gene transcriptional activity encoding this protein. In turn, analysis of ocular samples has been notoriously difﬁcult because of the technical obstacles. This is especially true for the lens and cornea [43]. Unfortunately, in our study we did not obtain sufficient quantities of the specimens for further analyses. Therefore, we could not measure the activity and level of other oxidative enzymes or metabolites, such as catalase or malondialdehyde, in this study. Although there are published data regarding differences between mRNA levels of antioxidants in the ocular tissues of patients with PEX [18,44,45], the identification of candidate genes as molecular markers of PEX in previous studies has not been performed.

Our results revealed statistically significant higher mRNA levels of *SOD2*, *ALDH1A1*, and *MGST1* in the ALCs of patients with PEX and cataract compared to the control cataract subjects suggesting that the pathways for regulating and responding to oxidative stress were present and transcriptionally responsive. Upregulation of *SOD2* may be a compensatory mechanism against free radicals, and an adaptive cellular response to increased production of ROS [46]. Previous studies indicated that SOD2 in the ocular tissues can be induced by mediators of oxidative stress, including radiation, lipopolysaccharide, tumor necrosis factor, and interleukin 1 [47,48]. Zenkel et al. [18,45] determined the set of differentially expressed genes mainly related to extracellular matrix metabolism and cellular stress in anterior segment tissues obtained from patients with PEX-associated open-angle or closed-angle glaucoma patients, at the mRNA and protein levels. Corresponding to our results, the expression of SOD increased in their studies. Furthermore, Uçakhan et al. [49] demonstrated an increase in SOD activity in the lens capsules of patients with PEX, but in that study, qRT-PCR was not used. This may suggest the compensatory mechanism in response to the increased oxidative stress in eyes with PEX and cataract compared to those with cataract alone [49]. Lin et al. [50] suggested that overexpression of SOD could prevent cataract formation induced by oxidative stress. However, they studied rat intact lenses. All these results suggested that SOD activity may play a role in eliminating oxidative stress.

MGST1 may be a protective factor against oxidative damage from lipid peroxidation [51]. In the current studies, a statistically significant increase in the *MGST1* mRNA level was revealed. This finding suggests that oxidative stress plays a role in the pathogenesis of PEX. However, the report presented by Zenkel et al. [45] contradicts our findings. These authors found up to threefold downregulation for *MGST1* in PEX-associated open-angle or closed-angle glaucoma specimens. In addition, Yağci et al. [42] observed a decrease in SOD activity. The discrepancy could be due to the patient pool; e.g., the patients in the Zenkel et al. [45] study had glaucoma comorbidity rather than cataract comorbidity. Reduced expression of antioxidative enzymes can indicate defective protection against oxidative stress in patients with PEX [42,45]. Yildirim et al. [52] and Yilmaz et al. [53] also suggested that decreased trace element levels in the lenses of patients with PEX could reflect defective antioxidative defense systems. Additionally, high concentrations of lipid peroxidation products may promote oxidation, aggregation, and abnormal extracellular matrix metabolism in the anterior segment structures of eyes and cause disease progression [54].

ALDH1A1 plays a role in oxidation of a wide range of aldehydes, e.g., all-trans- and 9-cis-retinal, and exhibits a high affinity for metabolism of highly reactive products of lipid peroxidation of cellular membranes, including malondialdehyde (MDA) and 4-hydroxy-2-nonenal [55]. Thus, ALDH1A1 plays a role in preventing oxidative damage by free-radical species [26,56,57]. In ocular tissues, ALDH1A1 is found mainly in the lens and to a lesser extent in the cornea. ALDH1A1 can play a role in detoxifying 4-hydroxy-2-nonenal and MDA, protecting ocular tissues from protein crosslinking and aggregation, which can lead to proteasome inhibition and eventually cataract formation [58]. Yilmaz et al. indicated that MDA concentrations were much higher in patients with PEX [38]. However, data are lacking regarding *ALDH1A1* expression in PEX. For the first time, our results showed increased expression of *ALDH1A1* gene in the ALCs of patients with this disease, which may be explained through the involvement of ALDH1A1 in the metabolism of vitamin A [59] and participation in the second and third lines of antioxidant defense [60].

The second part of this study focused on assessing *SOD2*, *MGST1*, and *ALDH1A1* mRNA levels as molecular markers of PEX development. While we were launching our study, we could not find any published reports regarding the identification of correlations between PEX and selected markers. Our findings suggest the clinical usefulness of these molecular markers for PEX. Moreover, the ALDH1A1 holds great potential for developing diagnostics for PEX because ROC analysis revealed sensitivity and specificity of 85.7% and 100%, respectively, with an AUC of 0.957 as an index of diagnostic accuracy. However, the suggestion that the level of *ALDH1A1* mRNA could be a good predictor of risk for PEX seems speculative. ALDH1A1 appears to be a good discriminator of controls and patients with PEX in a population with sufficiently advanced disease to allow for clinical diagnosis, but there is no evidence that *ALDH1A1* mRNA levels measured earlier in the progression of the disease will be as sensitive or accurate. A possible role of *ALDH1A1* mRNA level as a risk factor or marker for PEX requires further investigation. Moreover, our data are limited in that not the entire PEX population but only samples from patients with PEX and cataract were evaluated.

In conclusion, *SOD2*, *ALDH1A1*, and *MGST1* were overexpressed in the ALCs of patients with PEX suggesting that diseased tissue appears to respond to oxidative stress. Knowledge of the molecular pathophysiology of PEX may provide insight into the development of possible strategies for early diagnosis of PEX. Moreover, a detailed examination of the antioxidant defense system might help to identify a new therapeutic agent for PEX treatment.

## 

**Table:** 

			
